# Diagnostic accuracy of Mirels score, CT-based Structural Rigidity Analysis, and Finite Element Analysis for predicting impending femoral pathologic fractures in metastatic bone disease: a systematic review

**DOI:** 10.1016/j.jbo.2026.100790

**Published:** 2026-08-13

**Authors:** Bisola Salaja, Matthew Lee, Gary O'Toole, Alan Molloy

**Affiliations:** a Department of Trauma & Orthopaedics, St. Vincent's University Hospital, Dublin, Ireland; b University College Dublin, Dublin, Ireland

**Keywords:** Femoral metastases, Pathologic fracture, Fracture risk prediction, Mirels score, CT-based structural rigidity analysis

## Abstract

**Background:**

Patients with metastatic bone disease affecting the femur are at substantial risk of pathologic fracture, resulting in significant morbidity and frequently necessitating urgent surgical intervention. Accurate identification of lesions at imminent risk of fracture enables timely prophylactic fixation and improved patient outcomes. Although the Mirels score remains widely used, its limited specificity has prompted interest in CT-derived biomechanical approaches, including CT-based Structural Rigidity Analysis (CTRA) and Finite Element Analysis (FEA). Whole-femur CT is routinely obtained in some specialist orthopaedic oncology pathways, including our own, but this practice is not universal; many centres rely initially on clinical assessment and plain radiographs, with CT reserved for selected cases. We systematically reviewed the literature to compare evidence for Mirels, CTRA, and CT-based FEA in predicting pathologic femoral fractures.

**Methods:**

A systematic search of MEDLINE and Embase was performed for studies published between 2005 and 2025. Eligible studies evaluated Mirels scoring, CTRA, or CT-based FEA in adult patients with femoral metastatic disease and reported subsequent pathologic femoral fracture outcomes. Two reviewers independently screened studies and extracted data. Potential cohort overlap was assessed by comparing authorship, recruiting institutions, enrolment periods, eligibility criteria, and descriptions of prior datasets. Management after fracture-risk assessment was examined to identify intervention-related bias. Due to heterogeneity in study design, imaging methods, and outcome reporting, a qualitative narrative synthesis was undertaken.

**Results:**

Eight studies met the inclusion criteria, comprising prospective and retrospective clinical cohorts, an implementation study, and one illustrative comparative case series. In studies reporting formal diagnostic estimates, Mirels sensitivity ranged from 66.7% to 88%, while specificity ranged from 38% to 47.9%. In the principal prospective comparison, CTRA achieved 100% sensitivity and 60.6% specificity. Across formal FEA analyses, sensitivity ranged from 80% to 100% and specificity from 67% to 86%, although modelling methods, thresholds, and comparators varied. The evidence arose from small, partly overlapping cohorts, and prophylactic stabilization of lesions considered high risk limited observation of untreated outcomes and particularly the positive predictive value. Heterogeneity precluded meta-analysis.

**Conclusion:**

Mirels remains a useful, accessible, and sensitive first-line screening tool, particularly where plain radiography is the principal imaging modality. CTRA and CT-based FEA may offer additional specificity and objective mechanical information in selected patients where suitable calibrated CT imaging and technical expertise are available, but current evidence does not support replacing clinical assessment and radiography. Whole-femur CT is routine in some specialist pathways, including our own, but not across all centres. Further progress will require prospective multicentre validation, standardized methodologies, health-economic assessment, and integration within multidisciplinary team pathways.

## Highlights

• Mirels scoring is sensitive but lacks specificity for metastatic femoral fracture prediction.
• CTRA and CT-based FEA show improved discrimination compared with Mirels scoring.
• CT-based fracture-prediction tools align with modern whole-femur CT workflows.
• Prospective validation and MDT-linked metastatic bone registries are needed.

## 1 Introduction

Metastatic disease involving the femur carries a substantial risk of pathologic fracture, an event that can result in severe pain, loss of mobility, prolonged hospitalization, and urgent surgical intervention in patients who are often already systemically unwell. Accurate identification of femoral lesions at imminent risk of fracture is therefore critical to guide timely prophylactic fixation and avoid both catastrophic fracture and unnecessary surgery [1], [2].

Historically, the Mirels scoring system has been the most widely used clinical tool to assess fracture risk in long-bone metastases [3]. First described in 1989, Mirels proposed a simple additive score based on four variables—anatomic site, lesion type, lesion size, and pain severity—to guide decisions regarding prophylactic fixation. Although originally developed using plain radiographs, Mirels scoring has since been routinely applied to computed tomography (CT) imaging in contemporary clinical practice. The high sensitivity of the Mirels score supports its continued role as a screening tool, particularly given the potentially serious consequences of missing an impending pathologic fracture [2]; however, its specificity is limited, particularly for intermediate-risk lesions, which may lead to overtreatment and unnecessary surgical intervention [4], [5].

### 1.1 Mirels scoring system

The Mirels score assigns 1–3 points across four domains, with a total score ranging from 4 to 12. A score of ≥9 is commonly used to recommend prophylactic fixation [3].

**Table:** 

**Variable**	**1 point**	**2 points**	**3 points**
**Site**	Upper limb	Lower limb	Peritrochanteric region
**Pain**	Mild	Moderate	Functional pain
**Lesion type**	Blastic	Mixed	Lytic
**Size (% cortical involvement)**	< 1/3	1/3–2/3	> 2/3

At our institution, whole-femur CT is routinely used for suspected or known femoral metastases, although many centres still rely initially on clinical assessment and plain radiographs due to availability and cost. Where CT is obtained, it provides superior anatomic detail compared with plain radiography, and allows identification of additional femoral lesions and more accurate assessment of cortical involvement [6]. As a result, CT-based fracture prediction approaches are increasingly clinically relevant, scalable, and aligned with contemporary orthopaedic oncology pathways [7].

Two CT-derived biomechanical approaches have emerged as potential alternatives to conventional scoring systems: CT-based Structural Rigidity Analysis (CTRA) and CT-based Finite Element Analysis (FEA) [8]. CTRA uses CT imaging to assess bone shape and density to estimate how resistant a bone is to bending, torsion, and compression, providing an objective measure of strength loss compared with normal bone [9]. In contrast, CT-based FEA uses patient-specific three-dimensional models to simulate physiologic loading conditions and directly estimate failure risk based on predicted stress or strain distributions within the bone [10], [11].

Both CTRA and CT-based FEA aim to quantify structural failure risk more directly than clinical scoring systems. Early biomechanical and clinical validation studies suggest improved fracture-risk discrimination compared with Mirels scoring, and several CT-based tools—most notably CTRA and FE-derived decision-support scores—have undergone limited prospective clinical validation [7]. However, the evidence base for these techniques in metastatic femoral disease remains heterogeneous, with variation in study design, thresholds, and outcome definitions. Practical requirements—including access to suitable CT imaging, calibration, specialist software, and technical expertise—also limit generalizability. Importantly, this body of evidence has not been systematically synthesized in a manner that supports routine clinical adoption or informs future implementation pathways.

Therefore, the purpose of this study was to systematically review the available evidence comparing Mirels scoring, CT-based Structural Rigidity Analysis, and CT-based Finite Element Analysis for predicting pathologic femoral fractures in patients with metastatic bone disease, and to identify priorities for future research, validation, and clinical integration.

## 2 Methods

### 2.1 Study design and protocol

This systematic review was conducted in accordance with the Preferred Reporting Items for Systematic Reviews and Meta-Analyses (PRISMA) guidelines.

### 2.2 Eligibility criteria

A systematic search of MEDLINE (via PubMed) was performed using terms related to femoral anatomy, metastatic disease, fracture outcomes, and fracture-prediction tools. The search strategy was:

(femur OR femoral) AND (metastasis OR metastatic OR bone metastases) AND (Mirels OR “structural rigidity” OR CTRA OR “finite element” OR FEA) AND (fracture OR pathologic fracture OR impending fracture)

Limits applied were humans, English language, and publication years 2005–2025.

A separate search of Embase (via Ovid) was conducted. The search combined the following concepts using Boolean AND:

femur AND metastasis AND fracture AND (Mirels OR structural rigidity OR finite element).

Limits applied were humans, English language, and publication years 2005–2025.

### 2.3 Study selection

All retrieved records were imported into Rayyan (Qatar Computing Research Institute) for deduplication and screening. Two reviewers independently screened titles and abstracts for eligibility.

Data were extracted independently by two reviewers. Extracted variables included study characteristics, patient and lesion numbers, tumour origin, index test(s) evaluated, imaging and calibration methods, diagnostic thresholds, follow-up duration, reported accuracy measures, management following fracture-risk assessment, and the definition and occurrence of pathologic femoral fracture. A summary of the extracted data is provided in Table 2. Sample size was defined as the number of patients or femoral lesions included in the fracture-prediction analysis, rather than the total number initially enrolled. (See Table 1.)

**Table 1:** Eligibility criteria for study inclusion.

	Criteria
Inclusion criteria	Adult patients (≥18 years) with metastatic bone disease involving the femur
	Evaluation of at least one fracture-prediction tool: Mirels score; CT-based Structural Rigidity Analysis (CTRA); or CT-based Finite Element Analysis (FEA)
	Assessment based on plain radiographs and/or CT imaging
	Reported subsequent pathologic femoral fracture outcomes
	Prospective or retrospective clinical studies, including comparative clinical case series with extractable fracture outcomes
	Published from 2005 onward
	Published in English
Exclusion criteria	Cadaveric, phantom, or purely biomechanical studies without clinical fracture outcomes
	Studies focused on treatment optimization (e.g., femoroplasty) rather than fracture prediction
	Single-patient case reports or case series without comparative fracture-prediction data and subsequent fracture outcomes
	Studies without femur-specific data or without fracture outcomes
	Narrative reviews, systematic reviews, editorials, or conference abstracts without sufficient data
	Paediatric studies

### 2.4 Assessment of cohort overlap and management-related bias

Potential overlap between study populations was assessed by comparing authorship, recruiting institutions, enrolment periods, eligibility criteria, and references to previously reported datasets. Where overlap was confirmed or considered likely, it was recorded and considered in the qualitative interpretation. Management after risk assessment was also examined because prophylactic stabilization may prevent observation of the untreated fracture outcome, while exclusion of surgical referrals may remove the highest-risk lesions from analysis.

### 2.5 Data synthesis

Due to heterogeneity in study design, fracture definitions, diagnostic thresholds, comparators, and reporting of accuracy metrics, quantitative meta-analysis was not performed. Findings were synthesized narratively by fracture-prediction modality and at individual-study level. Numerical sensitivity, specificity, predictive values, and area-under-the-curve estimates were tabulated where reported.

### 2.6 Outcomes

The primary outcome was subsequent pathologic femoral fracture occurring after assessment with the index test. Secondary outcomes included reported diagnostic accuracy measures, time to fracture, and implications for clinical decision-making where available.

## 3 Results

### 3.1 Study selection

**Fig. 1:**
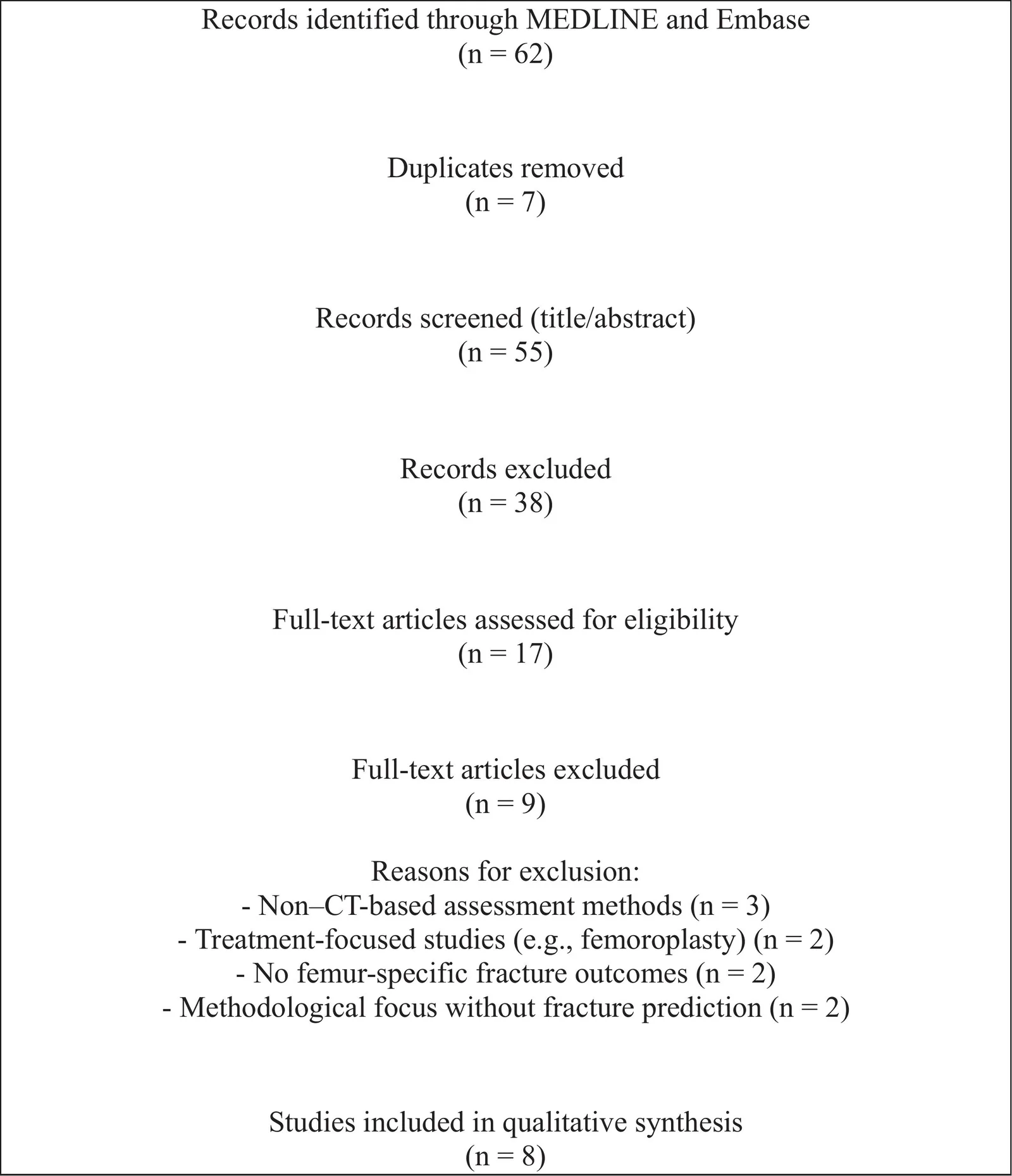
PRISMA flow diagram of study identification, screening, eligibility assessment, and inclusion.

Searches of MEDLINE and Embase identified 62 records, of which 7 duplicates were removed. Following title and abstract screening of 55 records, 38 studies were excluded, most commonly due to irrelevant outcomes, inappropriate populations, or unsuitable study designs, including cadaveric or biomechanical studies focused on treatment optimization rather than fracture prediction. Seventeen full-text articles were assessed for eligibility, of which eight studies met the inclusion criteria and were included in the final qualitative synthesis (Fig. 1).

### 3.2 Study characteristics

The characteristics of the included studies are summarized in Table 2. The eight included studies were published between 2015 and 2022 and represented prospective diagnostic cohorts, retrospective analyses, a prospective implementation cohort, and one illustrative comparative case series. Studies were conducted in the United States, Israel, and the Netherlands.

**Table 2:** Characteristics of included studies.

First author (year)	Journal	Country	Study design	Sample size (analysed)	Tumour origin (descending prevalence)	Index test(s)	Imaging modality	Follow-up	Potential cohort overlap
Damron (2016)	Clin Orthop Relat Res	USA	Prospective multicentre diagnostic cohort	78 lesions (125 enrolled)	Mixed metastatic primaries	CTRA, Mirels	XR, quantitative CT	12 months	North American/MSTS programme; potential overlap with Goodheart and Kaupp
Goodheart (2015)	J Orthop Res	USA	Prospective cohort analysis	38 patients / 44 femurs	Breast, lung, multiple myeloma, prostate, renal	CT-based FEA	XR, CT	4 months	Same North American research programme; likely partial overlap
Eggermont (2018)	Bone & Joint Research	Netherlands	Prospective multicentre cohort	39 patients	Mixed metastatic primaries	CT-based FEA	Quantitative CT	6 months	Potential partial overlap with Eggermont 2020
Sternheim (2018)	Bone	Israel	Retrospective cohort	50 patients	Breast, multiple myeloma, other, lung, renal	CT-based FEA, Mirels	CT	5 months for untreated subgroup	Same centre and related programme as Sternheim 2020; overlap possible
Sternheim (2020)	Bone & Joint Journal	Israel	Retrospective cohort	82 patients; diagnostic comparison *n* = 33	Breast, multiple myeloma, other, lung, renal	CT-based FEA, Mirels	CT	5–6 months	Same centre and related programme as Sternheim 2018; overlap possible
Eggermont (2020)	Bone	Netherlands	Prospectively enrolled cohort analysis	45 patients / 50 lesions	Lung, breast, other, multiple myeloma, prostate	CT-based FEA	XR, quantitative CT	6 months	Related Dutch cohorts; potential partial overlap with Eggermont 2018
Eggermont (2022)	Cancers	Netherlands	Prospective implementation cohort	39 patients / 42 femurs	Breast, lung, other, prostate, melanoma	CT-based FEA (BOS score)	CT	6 months	Later implementation cohort; no clear overlap reported
Kaupp (2021)	Advances in Orthopaedics	USA	Illustrative comparative case series	7 patients / 8 femurs	Mixed metastatic primaries	Mirels, CTRA, CT-based FEA	XR, CT	Up to 12 months	Selected from a larger North American dataset; overlaps with the research programme

All studies included adult patients with metastatic bone disease involving the femur and reported subsequent pathologic femoral fracture outcomes during a defined follow-up period, which ranged from 4 to 12 months. Sample sizes varied from small case-based cohorts to 82 patients, with several studies reporting both patient- and lesion-level analyses. Tumour origin was heterogeneous across studies, most commonly including breast carcinoma, lung carcinoma, multiple myeloma, prostate carcinoma, renal cell carcinoma, and melanoma.

All included studies used CT-based imaging, either exclusively or in combination with plain radiographs, reflecting contemporary diagnostic pathways.

### 3.3 Fracture-prediction tools

#### 3.3.1 Mirels score

The Mirels score was evaluated in five studies, either alone or in direct comparison with CT-based techniques. In the prospective Damron cohort, Mirels ≥9 had sensitivity of 66.7%, specificity of 47.9%, positive predictive value (PPV) of 9.8%, and negative predictive value (NPV) of 94.4% [9]. Goodheart et al. reported sensitivity of 80% and specificity of 43% [12], while Sternheim et al. reported sensitivity of 88%, specificity of 38%, and an area under the curve (AUC) of 0.578 [6]. Overall, Mirels remained a sensitive screening tool but showed limited specificity, with substantial false-positive classification. In direct comparisons, Mirels was less effective than CT-based methods at distinguishing lesions that subsequently fractured from those that did not, particularly in intermediate-risk cases [8], [9], [12], [13].

#### 3.3.2 CT-based Structural Rigidity Analysis (CTRA)

CTRA was evaluated in one prospective multicentre cohort and one illustrative comparative case series [8], [9]. Using a threshold of at least 35% reduction in axial, bending, or torsional rigidity, Damron et al. reported sensitivity of 100%, specificity of 60.6%, PPV of 17.6%, and NPV of 100%, compared with 66.7%, 47.9%, 9.8%, and 94.4%, respectively, for Mirels ≥9 [9]. The confidence intervals overlapped substantially, and the low PPV reflected the low number of observed fractures. Kaupp et al. illustrated concordant and discordant Mirels, CTRA, and FEA classifications in eight femurs but was not designed to generate formal diagnostic-accuracy estimates [8].

#### 3.3.3 CT-based Finite Element Analysis (FEA)

CT-based FEA was evaluated in seven included reports, although the modelling methods, loading conditions, thresholds, and comparators differed [6], [8], [12], [13], [14], [15], [16]. Goodheart et al. reported sensitivity of 80% and specificity of 86% for level-walking FEA, compared with 80% and 43% for Mirels; performance varied markedly with the simulated loading condition [12]. Eggermont et al. reported sensitivity of 89% and specificity of 79%, compared with clinician sensitivity of 0%–33% and specificity of 84%–95% [15]. Sternheim et al. subsequently reported sensitivity of 100%, specificity of 67%, and AUC of 0.905 for the strain fold ratio, compared with 88%, 38%, and 0.578 for Mirels [6]. In a separate cohort, Eggermont et al. reported FEA sensitivity of 100%, specificity of 74%, PPV of 39%, and NPV of 100%, compared with 86%, 42%, 19%, and 95%, respectively, for axial cortical involvement [14].

More recent work extended FEA beyond diagnostic prediction. In the BOS implementation study, 42 scores were delivered for 39 patients, and provision of the score led to adaptation of the treatment plan in 48% of assessments [16]. This study primarily examined feasibility and clinical decision support rather than formal diagnostic accuracy, and it should therefore not be weighted equivalently to prospective prediction cohorts.

### 3.4 Quantitative and study-level synthesis

The principal numerical findings, comparator methods, and study-specific limitations are summarized in Table 3. The included publications differed substantially in purpose: Damron et al. provided prospective head-to-head evidence for CTRA and Mirels; Goodheart, Sternheim, and Eggermont evaluated distinct FEA methods; Eggermont 2022 assessed clinical implementation; and Kaupp provided an illustrative case series. The reports were therefore interpreted individually rather than as a uniform set of comparative diagnostic studies.

**Table 3:** Quantitative findings and principal limitations of included studies.

Study	Evaluable cohort/outcome	Comparator	Principal quantitative findings	Key interpretive limitation
Damron (2016)	78 lesions; 12-month fracture outcome	CTRA vs Mirels ≥9	CTRA: sensitivity 100%, specificity 60.6%, PPV 17.6%, NPV 100%. Mirels: 66.7%, 47.9%, 9.8%, and 94.4%, respectively.	Only lesions without prophylactic stabilization and with sufficient follow-up were analysed; few fractures and wide/overlapping confidence intervals.
Goodheart (2015)	38 patients / 44 femurs; 5 fractured, 28 non-fractured, 11 stabilized	FEA vs Mirels	Level-walking FEA: sensitivity 80%, specificity 86%. Mirels: sensitivity 80%, specificity 43%. Performance differed by loading condition.	Stabilized femurs lacked an untreated fracture outcome; small number of fractures.
Eggermont (2018)	39 patients; 9 fractures in 7 patients	FEA vs experienced clinicians	FEA: sensitivity 89%, specificity 79%. Clinician assessment: sensitivity 0%–33%, specificity 84%–95%.	Prospective radiotherapy cohort; inter-scanner differences and small fracture count.
Sternheim (2018)	50 patients; 11 untreated after surgical referral	CTFEA threshold; clinical referral	Among 11 untreated patients, none fractured within 5 months; CTFEA classified 7 as low risk and 4 as high risk, giving specificity 63%.	Threshold derived from 5 pre-fracture cases; treatment-selection and verification bias prevented robust sensitivity/PPV estimation.
Sternheim (2020)	82 total; 41 not prophylactically fixed; diagnostic comparison n = 33	CTFEA strain fold ratio vs Mirels	CTFEA: sensitivity 100%, specificity 67%, AUC 0.905. Mirels: sensitivity 88%, specificity 38%, AUC 0.578.	Retrospective selection and possible overlap with the earlier Israeli cohort.
Eggermont (2020)	45 patients / 50 femurs; 7 fractures	FEA vs axial cortical involvement	FEA: sensitivity 100%, specificity 74%, PPV 39%, NPV 100%. Axial cortical involvement: 86%, 42%, 19%, and 95%, respectively.	Radiotherapy-selected cohort may not represent patients already referred for prophylactic fixation.
Eggermont (2022)	39 patients / 42 femurs	BOS-assisted vs pre-BOS treatment plan	The BOS result led to adaptation of the treatment plan in 48% of assessments.	Implementation study rather than a formal independent diagnostic-accuracy study; management was influenced by the index result.
Kaupp (2021)	7 patients / 8 femurs	Mirels, CTRA, and FEA	Descriptive examples of true-positive, true-negative, false-positive, and false-negative classifications; no formal summary accuracy estimates.	Illustrative selected cases from a larger dataset; not an independent diagnostic cohort.

### 3.5 Potential overlap between cohorts

Potentially overlapping populations were identified within three research programmes. Goodheart and Kaupp arose from the North American research programme that also underpinned the multicentre Damron study, and Kaupp explicitly selected illustrative femurs from a larger dataset [8], [9], [12]. Eggermont 2018 and Eggermont 2020 used related Dutch prospective cohorts and may include partial overlap [14], [15]. Sternheim 2018 and Sternheim 2020 originated from the same Israeli centre and related CTFEA programme, with possible overlap that could not be quantified from the published reports [6], [13]. Accordingly, the eight publications represent fewer than eight fully independent patient populations.

### 3.6 Management pathways and verification of fracture outcome

Management after risk assessment varied and affected verification of the fracture outcome. In Damron et al., 78 of 125 enrolled lesions did not undergo prophylactic stabilization and had sufficient follow-up for the primary analysis [9]. Goodheart et al. analysed 5 fractured and 28 non-fractured femurs while reporting 11 stabilized femurs separately [12]. In Sternheim 2018, most patients had been referred for prophylactic surgery; among 11 who did not undergo surgery, none fractured during the designated five-month follow-up, allowing specificity but not sensitivity to be estimated in that subgroup [13]. The Dutch cohorts predominantly included patients receiving palliative radiotherapy [14], [15] while the BOS result itself altered treatment planning in 48% of cases [16]. These pathways introduce intervention and spectrum bias and make PPV particularly difficult to interpret.

## 4 Discussion

The available studies suggest that CTRA and selected CT-based FEA methods may improve specificity or discrimination compared with Mirels in some metastatic femoral cohorts. However, the evidence does not support replacing clinical assessment and plain radiography. Mirels retains important clinical utility because it is accessible, inexpensive, incorporates symptoms, and prioritizes sensitivity in a setting where failure to identify an impending fracture may have serious consequences. The apparent advantages of CT-based methods must also be interpreted in the context of small and partly overlapping cohorts, heterogeneous methods, and intervention-related bias.

### 4.1 Mirels score: strengths and limitations

Since its introduction in 1989, the Mirels score has been widely adopted due to its simplicity and ease of use [3]. In this clinical context, some overestimation of risk may be preferable to missing a lesion that subsequently fractures. Its limited specificity therefore represents, in part, a safety-oriented trade-off rather than evidence that the score lacks clinical value. However, multiple studies have demonstrated poor specificity and substantial interobserver variability, particularly for pain assessment and estimation of lesion size [2], [17]. Mirels should remain a first-line screening tool, but its result should be integrated with the overall clinical picture rather than used as an isolated indication for prophylactic fixation.

The anatomic-location component of Mirels also warrants scrutiny. In the original analysis, location did not independently improve fracture prediction despite being retained within the score [3]. Within a femur-specific population, every lesion receives at least two location points, while a peritrochanteric lesion receives three points regardless of its more precise axial or circumferential position. A modified location component informed by finite-element modelling reduced false-positive classifications by approximately 17%–20% in a clinical development cohort, although external validation is required before routine adoption [18].

### 4.2 CT-based Structural Rigidity Analysis

CTRA represents a biomechanical approach that quantifies loss of bone rigidity using CT-derived geometry and density. In the landmark prospective study [9], CTRA demonstrated superior specificity while maintaining 100% sensitivity compared with Mirels ≥9. Importantly, CTRA reduced false-positive fracture predictions while maintaining clinically acceptable sensitivity, suggesting improved patient selection for prophylactic fixation [8]. Despite promising validation, the validated workflow required CT of both femurs with a density-calibration phantom, dedicated processing, and access to appropriate normative comparison data. Calibration phantoms are not routinely included in standard clinical CT protocols, and scanner and reconstruction differences may affect density and rigidity estimates. Phantomless or equivalent-density calibration methods have been developed for femoral quantitative CT in other settings but require validation specifically in metastatic femoral disease before they can remove this practical barrier [19].

### 4.3 CT-based Finite Element Analysis

CT-based FEA was the most frequently evaluated modality in this review and demonstrated consistent improvement in fracture-risk discrimination across retrospective and prospective cohorts. Patient-specific FE models more accurately distinguished lesions that subsequently fractured from those that did not across multiple retrospective and prospective cohorts [12], [13], [14], [15]. Recent studies have also focused on translation into clinical practice, including the development of FE-derived decision-support tools such as the Bone Strength (BOS) score [16], demonstrating feasibility within routine multidisciplinary care pathways. However, FEA requires segmentation, density-to-material-property conversion, definition of boundary and loading conditions, and a failure criterion. Variation in these modelling choices, along with differences in software and calibration, limits direct comparison and reproducibility.

### 4.4 CTRA versus FEA

CTRA and FEA offer different potential advantages, and the present evidence does not establish that either is clinically superior. CTRA is comparatively simpler and produces readily interpretable measures of structural rigidity, while FEA provides a more detailed three-dimensional representation of loading, strain, and failure location. Conversely, FEA is more computationally demanding and sensitive to modelling assumptions, whereas CTRA relies on calibrated CT acquisition and simplified beam-theory assumptions. In an experimental comparison using femurs with simulated lytic defects, both methods correlated with measured failure load and no significant difference in prediction accuracy was demonstrated; FEA was slightly more accurate at an individual level, while CTRA could be performed more expediently by non-expert operators [10]. Prospective head-to-head clinical comparison is still required.

### 4.5 Integration of pain and clinical assessment

Unlike Mirels, CTRA and FEA do not directly incorporate pain. This distinction is clinically important because pain may reflect biological progression, microfracture, loading intolerance, or other factors not fully captured by structural modelling. In a prospective treatment-planning study, CTRA findings, pain, and primary tumour source were independently associated with the post-CTRA management plan, indicating that mechanical and clinical information are complementary rather than interchangeable [20]. No externally validated composite algorithm combining pain with CTRA or FEA was identified. Future prediction models should evaluate the incremental value of integrating mechanical strength with pain, functional loading, tumour biology, expected survival, and patient preferences.

### 4.6 Relevance to modern clinical practice

Imaging practice varies between centres. At our institution, whole-femur CT is routinely obtained in the assessment of femoral metastatic disease, making CT-derived analysis potentially compatible with the existing pathway. This should not be generalized to all settings. Plain radiographs remain simpler, more widely available, and less costly, and CT is not required for every patient. The most realistic near-term role for CTRA or FEA may therefore be as an adjunct in selected or equivocal cases when a suitable CT has already been obtained for clinical reasons. Formal health-economic evaluation is needed before advocating broader CT acquisition solely for biomechanical fracture-risk analysis.

### 4.7 Application beyond the femur

Related biomechanical approaches have been explored in spinal metastatic disease. In a prospective study of 94 patients with 247 metastatic vertebral lesions, CTRA-derived measures were 100% sensitive, with specificity ranging from 44% to 70% depending on the parameter and normalization method [21]. FEA has also been used to model the influence of vertebral tumour size, bone density, and loading on metastatic vertebral failure, but much of this evidence remains computational or experimental rather than validated for routine clinical decision-making [22]. These findings demonstrate broader biomechanical applicability but should not be directly extrapolated from the femur because vertebral loading, neurological consequences, and treatment thresholds differ substantially.

### 4.8 Limitations

The included studies were heterogeneous in design, follow-up duration, fracture definitions, comparators, calibration methods, and biomechanical thresholds, precluding meta-analysis. Sample sizes were modest, and several publications arose from overlapping or extended institutional cohorts, reducing independent replication. Not all reports were direct comparisons of Mirels, CTRA, and FEA. Prophylactic stabilization of lesions judged high risk prevented observation of their untreated natural history, while radiotherapy-focused cohorts may have excluded the highest-risk surgical referrals; these pathways introduce intervention, verification, and spectrum bias and limit estimation of PPV. Death was also an important competing event. Finally, the requirement for suitable CT imaging, calibration, software, and technical expertise limits generalizability to centres where these resources are unavailable.

### 4.9 Future directions

Future work should focus on adequately powered prospective multicentre studies with transparent assessment of cohort independence, standardized CT acquisition and calibration, harmonized biomechanical thresholds, and clearly defined fracture outcomes. Head-to-head comparison of CTRA and FEA is needed, together with external validation of composite models that incorporate pain and other clinical factors. Studies should predefine how prophylactic stabilization and death will be handled analytically, report health-economic outcomes, and evaluate implementation within multidisciplinary pathways and metastatic bone registries.

## 5 Conclusions

Mirels remains a clinically useful, accessible, and sensitive first-line screening tool for metastatic femoral lesions. CTRA and CT-based FEA may improve specificity and provide complementary objective biomechanical information in selected patients, but current evidence is limited by heterogeneous methods, overlapping cohorts, treatment-related bias, and practical implementation requirements. Whole-femur CT is routine in some specialist pathways, including our own, but is not universal; CT-based techniques should therefore be viewed as promising adjuncts rather than established replacements for clinical assessment and plain radiography.

## CRediT authorship contribution statement

**Bisola Salaja:** Writing – review & editing, Writing – original draft, Visualization, Software, Methodology, Investigation, Formal analysis, Data curation, Conceptualization. **Matthew Lee:** Writing – review & editing, Supervision. **Gary O'Toole:** Writing – review & editing, Supervision. **Alan Molloy:** Writing – review & editing, Supervision.

## Declaration of competing interest

The authors declare that they have no known competing financial interests or personal relationships that could have appeared to influence the work reported in this paper.
